# Aridity may alter the contributions of plants and fungi to grassland functions

**DOI:** 10.1371/journal.pbio.3002765

**Published:** 2024-08-15

**Authors:** Julie R. Deslippe

**Affiliations:** School of Biological Sciences, and Centre for Biodiversity and Restoration Ecology, Te Herenga Waka–Victoria University of Wellington, Wellington, New Zealand

## Abstract

Grassland aridification threatens biodiversity which supports ecosystem multifunctionality (EMF), but the relative roles of biota in maintaining EMF are poorly known. This Primer explores a new study in PLOS Biology that finds complementarity of above- and below-ground biodiversity and a trade-off between fungal and plant richness in driving EMF with aridity.

## Teaser

Grassland aridification threatens biodiversity which supports ecosystem multifunctionality (EMF), but the relative roles of biota in maintaining EMF are poorly known. A new study in *PLoS Biology* finds complementarity of above- and belowground biodiversity and a trade-off between fungal and plant richness in driving EMF with aridity.

**Fig 1:**
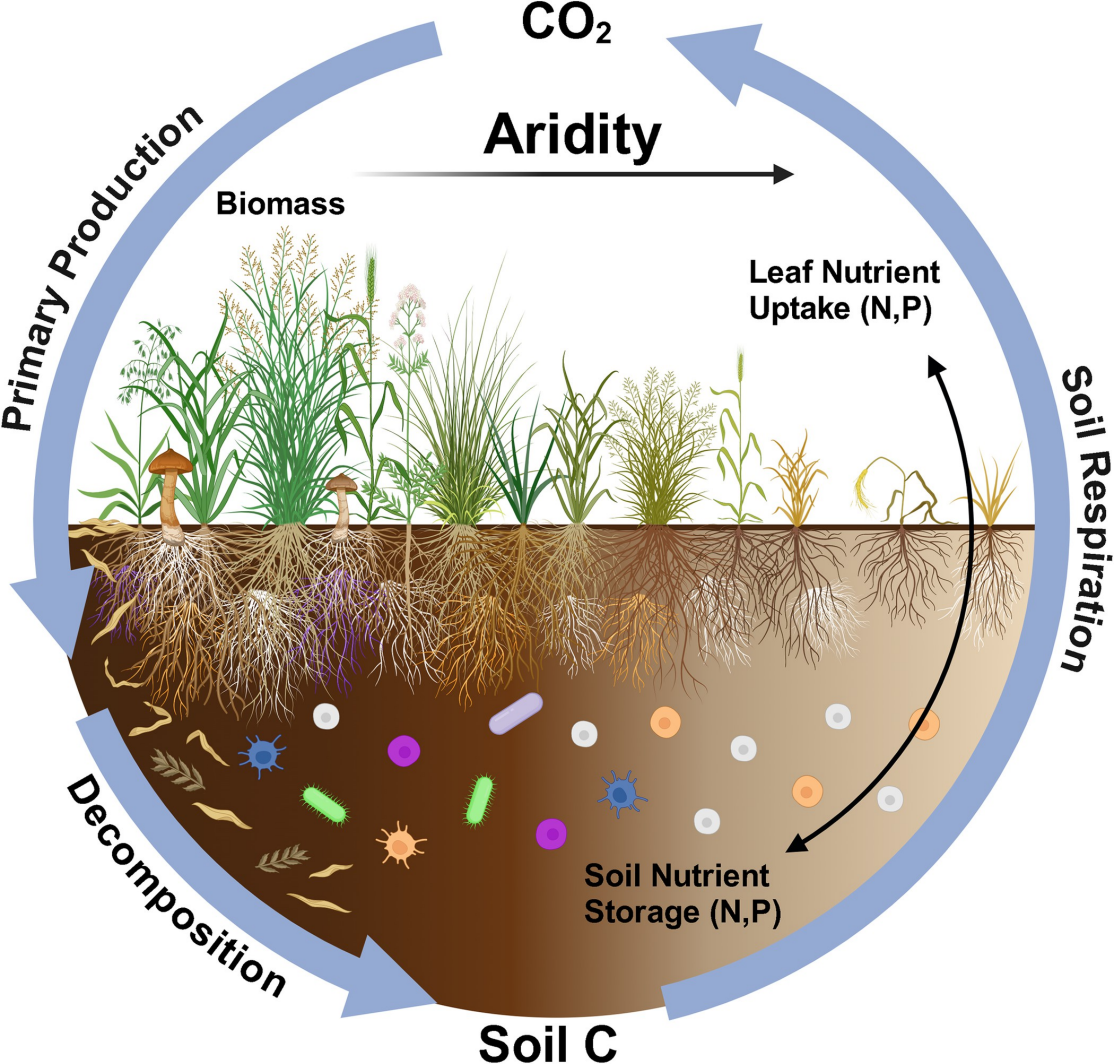
Grassland plant and soil microbial diversity drive ecosystem multifunctionality across a gradient of aridity. Ecosystems functions include carbon and nutrient cycling, which drive ecosystems services to people, such as carbon sequestration and nutrient storage. Primary production fixes atmospheric carbon and soil nutrients in above- and belowground plant biomass. Soil carbon stocks are built up via plant litter and root exudation to form soil organic matter. Soil organic matter stores nutrients and fuels a complex web of fungi, bacteria, and other life that decompose C-rich compounds and release CO_2_ back to the atmosphere through respiration, mineralising soil nutrients in the process. In this issue of *PLoS Biology*, Martins and team [7] reveal complementary links between above- and belowground biodiversity and a trade-off between fungal and plant richness in driving EMF with aridity. Created with BioRender.com.

The actions and interactions of species in nature affect ecosystem functions (e.g., carbon and nutrient cycling) that generate services (e.g., carbon sequestration, water purification) upon which humanity depends (**Fig 1**). Links between biodiversity and ecosystem function have fascinated ecologists for decades, with grasslands providing important study systems (e.g., [1]). While early studies focused on single ecosystem functions, increased recognition of the multiple functions and services simultaneously provided by ecosystems has led inquiry to shift toward a more integrated assessment of ecosystem multifunctionality (EMF, [2]). This change, concomitant with increased understanding of human-driven global biodiversity declines, has motivated a new generation of ecological studies. These seek understanding of the complementarity and redundancy of multitrophic communities in delivering EMF, particularly in the context of key drivers of ecosystem change such as increased CO_2_ [3], warming [4], and drought [5]. Essentially, these studies ask: “how much biodiversity loss can nature endure, before people start to feel it?” Alongside empirical studies, observational studies have generated essential insights. For example, Jing and colleagues [6] showed that regional-scale variation in climate modifies the effects of biodiversity on EMF, with soil moisture a key driver of this change.

In this issue, Martins and colleagues [7] contribute a further advance to our understanding of how moisture stress alters the relative contributions of biodiversity to EMF. They place their study in the context of grassland aridification, the progressive drying that affects more than 40% of lands globally. Rainfall deficit and climate warming lead to drought (i.e., prolonged soil moisture deficit), exacerbating inappropriate land-use and driving biodiversity losses in grasslands. However, we still know little of how these changes, both above- and belowground, alter grassland EMF globally. They tackle this question by measuring EMF in an impressive array of 101 globally distributed grasslands and also in a large-scale drought mesocosm study.

In the global survey, they elucidate the shared and unique contributions of plant and soil microbial diversity in supporting EMF across 101 grasslands. They find high correlations between plant and multitrophic richness and EMF, with about two thirds of the explained variance attributable to shared plant and microbial richness, while an additional third was attributed to plant richness alone. Likewise, about half of the variation in plant production across grasslands was attributed to microbial richness and shared plant and microbial richness, agreeing with previous studies indicating complementary links between above- and belowground diversity in delivering EMF [2]. Using structural equation modelling, the authors resolve the direct positive effects of plant and fungal richness on EMF, as well as those abiotic effects that are mediated by plant richness. Finally, they reveal a novel trade-off between fungal and plant richness in driving variation in EMF across the aridity gradient.

Next, Martins and colleagues employ a large-scale glasshouse study, wherein they construct potted plant–soil communities (mesocosms) that vary in plant and soil microbial diversity. Following exposure to drought, the authors measured a range of ecosystem functions in the mesocosms, comparing treatment groups to controls. They find strong correlations between plant, fungal, and multitrophic richnesses and EMF. While the total variance explained of EMF and all individual ecosystem functions and services was low, results of the mesocosm study broadly corroborate findings from their global survey, with roughly a third of the variance explained in EMF attributable to plant or shared plant and microbial richness. Likewise, multitrophic richness was associated with higher levels of function in a greater number of functions compared with the richnesses of single groups, again supporting the view that multitrophic richness maximises EMF under drought. A strength of the mesocosm data analysis is the partitioning of plant richness and plant composition as predictors of EMF. Plant composition of mesocosms explained slightly more variance in EMF than plant richness, suggesting that plant traits were important determinants of EMF. Further, structural equation modelling revealed only direct and independent effects of plant richness, plant composition, and microbial richness in driving ecosystem functions in the mesocosms. Accordingly, the authors stress the unique and complementary roles of these components of diversity in driving EMF under drought.

Aridification compounds unsustainable land-use practices and contributes to a progressive decline of grasslands through a sequence of vegetation loss, soil disruption, and desertification [8]. While reductions in above- and belowground biodiversity are associated with each stage of this decline, they are not inevitable, but can be reversed through effective mitigation and restoration strategies, which include improved livestock and fire management and the promotion of drought tolerant native plant species [9,10]. The paper by Martins and colleagues [7] enhances our understanding of the relative contributions of plant and soil biodiversity to maintaining EMF in an increasingly arid world and points to key taxonomic groups to monitor for decline. To adequately inform sustainable grassland management, our next steps must include improved understanding of the trajectories of these communities following restoration actions, only then can we ensure the many benefits that people derive from grasslands.
